# The Clinical Relations of Ataxy

**Published:** 1899-05-13

**Authors:** 


### THE CLINICAL RELATIONS OF ATAXY.

In a clinical lecture delivered at the Queen Square Hospital, Dr. Ormerod,[1] after describing the various kinds and degrees of ataxy met with in tabes, went on to mention the other diseases in which ataxy may also be met with. First comes peripheral neuritis, in some cases of which there is a very close similarity to tabes. There may be the ataxic walk, the pain in the legs, and the absence of knee-jerk; but there is never the Argyll Robertson pupil. The pupil phenomenon is therefore very important in the differential diagnosis of these two diseases. In most cases of peripheral neuritis the disease develops rather quickly, whereas tabes almost always comes on slowly. Showing a case in which there was a curious mode of progression which might be confounded with ataxy, although not truly ataxic at all, but rather a high-stepping gait due to paralysis of the anterior muscles of the calf, Dr. Ormerod said: This woman has peculiar gait which does not consist in unsteadiness or in flourishing the feet, but in lifting the knees unduly high. Such a gait as that might give rise to confusion if you are not familiar with it. Her knee-jerk is absent, and there is no particular atrophy of muscle. It also happens that her present condition has developed very slowly, which again heightens its resemblance to tabes. Her pupil, however, reacts to light.

Another form of disease in which there may be the ataxic gait is disseminated sclerosis. Not that it is very common to see the subjects of disseminated sclerosis walk with a truly ataxic gait, a paraplegic gait being more frequently met with in that disease. In a case mentioned, however, although the patient, who had a very typical disseminated sclerosis, walked in an ataxic way, his knee-jerks were exaggerated, and he had typical nystagmus, tremor, and affection of speech, so that there was not much risk of confusion with tabes.

Another condition in which there may be ataxy is cerebellar disease, but here the gait is not quite the same as in tabes. A cerebellar patient staggers, but he rarely flourishes his feet. In the early stage it is conceivable that the two diseases might be confounded; but in cerebellar disease there is no impairment of sensation, and the pupils usually react to light. The knee-jerk may or may not be absent.

There are certain diseases in which not only the posterior columns of the cord are degenerated, but the lateral columns also. These diseases may be described anatomically as postero-lateral sclerosis, or combined sclerosis; and they may give rise to ataxy, and may, therefore, possibly be mistaken for tabes. This combined sclerosis occurs in a good many diseases which clinically are not very much alike; for example, in general paralysis of the insane, in ataxic paraplegia, and in hereditary ataxy or Friedreich's disease.

In general paralysis of the insane the principal and most striking lesion is in the brain, and usually the most obtrusive symptoms are mental. But there are certain cases in which the spinal cord is involved, the posterior and lateral columns being degenerated and the mental symptoms not very marked. In such cases there is risk of confusion with tabes.

In ataxic paraplegia the gait is not altogether ataxic and not altogether paraplegic, but a kind of mixture of the two, the ataxic or the paralytic characters predominating according as the posterior or the lateral columns are principally involved.

In Friedreich's disease the posterior columns are involved from the first, but we do not know when the lateral columns are attacked. The family history in these cases is important because we generally find that several members of the family are diseased, which is not the case in any of the diseases previously mentioned. The first symptom is usually ataxy, beginning in the legs and slowly spreading to the arms. The shooting pains so characteristic of tabes are not such a prominent or early feature as in that condition; very often they do not occur at all.

Then a symptom which is common in Friedreich's disease, but is not found in ordinary tabes, is deformity of the feet. The foot is humped up with a high plantar arch, and the toes are much drawn up, a condition which is due partly to paralysis of the interossei, and partly to contraction of other muscles. The curling up of the toes is generally the commencement of this deformity.

The knee-jerks are nearly always absent, even in the very early stage of this disease. Affection of speech is fairly constant in this disease, but it never occurs in tabes; it is a thick elisive sort of speech, the syllables being run together rather than disjointed as in tabes. Kyphosis and scoliosis of the spine are also apt to occur in Friedreich's disease. When a case is seen in an advanced stage there should be no danger whatever of confusion with tabes; nor, indeed, should there in the early stages, for it generally runs in families, it occurs in young people, and the sensory symptoms which are characteristic of tabes—pains, anæsthesia, and the like—are usually completely absent; and, of course, when there is deformity of the feet and spine, that clinches the diagnosis. Another symptom which occurs in this disease, although late as a rule, and which does not occur in tabes, is nystagmus.

[1] Clinical Jour., April 5.

